# Supplementary material for: A novel mobile genetic element with virus-like characteristics is widespread in the world’s oceans
Source: bioRxiv. 2025 Aug 3:2025.08.03.668216. Preprint. [Version 1] doi: 10.1101/2025.08.03.668216 (PMC12324425; doi:10.1101/2025.08.03.668216)
Supplement: Supplement 5 [file NIHPP2025.08.03.668216v1-supplement-5.pdf]

bioRxiv preprint doi: https://doi.org/10.1101/2025.08.03.668216; this version posted August 3, 2025. The copyright holder has placed this preprint (which was not certified by peer review) in the Public Domain. It is no longer restricted by copyright. Anyone can legally share, reuse, remix, or adapt this material for any purpose without crediting the original authors.

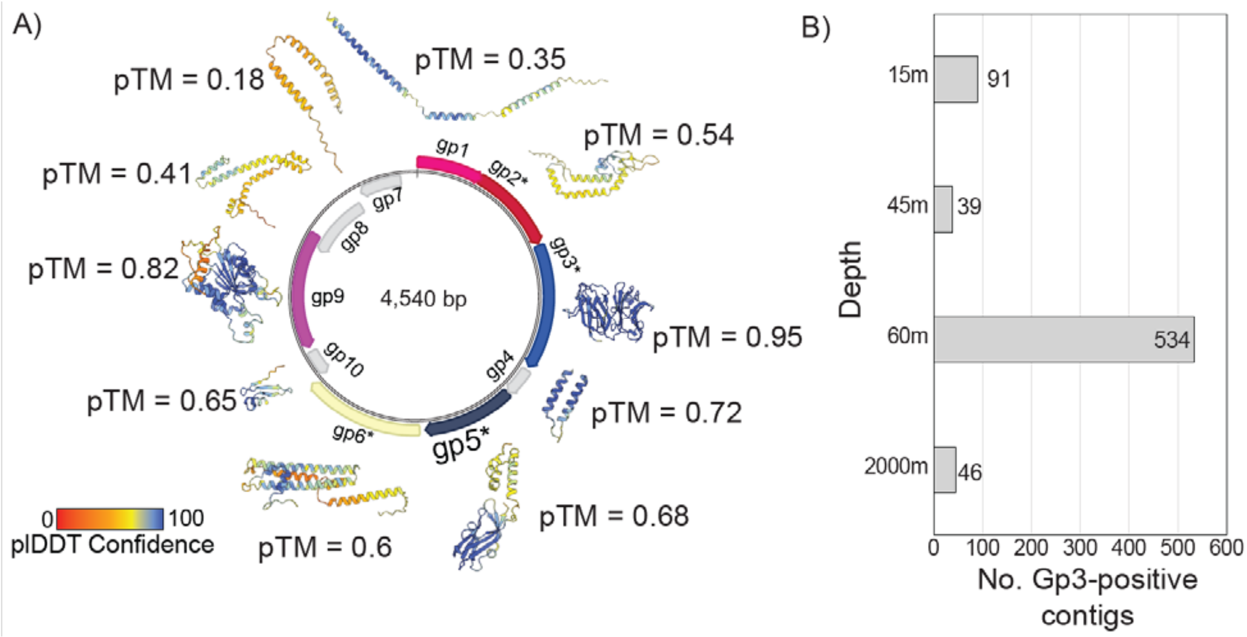

Figure S1: Structural predictions and depth distribution for Gp3-positive MAGs. a) Alphafold3 structure predictions of proteins encoded in an exemplary MAG. Arrows on circular genome indicate predicted genes, with asterisks indicating clear gene conservation across multiple genomes. Alphafold3 pTM score represents the predicted template modeling confidence, with > 0.5 suggesting that the predicted complex might be similar to the true structure. pLDDT (predicted local distance difference test) represents a per-atom confidence score of predictions, with 100 (blue) representing the highest confidence and 0 (red) the lowest. B) Number of contigs encoding Gp3 in mediterranean sea virome PRJEB30684.

bioRxiv preprint doi: https://doi.org/10.1101/2025.08.03.668216; this version posted August 3, 2025. The copyright holder has placed this preprint (which was not certified by peer review) in the Public Domain. It is no longer restricted by copyright. Anyone can legally share, reuse, remix, or adapt this material for any purpose without crediting the original authors.

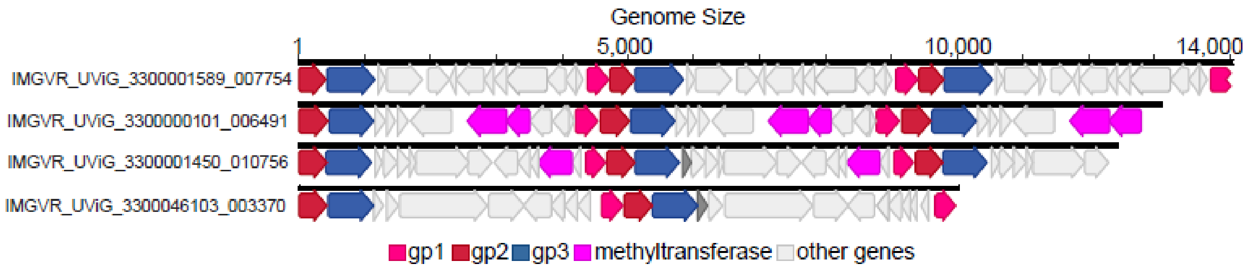

Figure S2: Examples of Charybdis genome concatemers in the IMGVRv4 database. Arrows indicate genes, gp2 was chosen as an arbitrary 5’ end of the genomes.

bioRxiv preprint doi: https://doi.org/10.1101/2025.08.03.668216; this version posted August 3, 2025. The copyright holder has placed this preprint (which was not certified by peer review) in the Public Domain. It is no longer restricted by copyright. Anyone can legally share, reuse, remix, or adapt this material for any purpose without crediting the original authors.

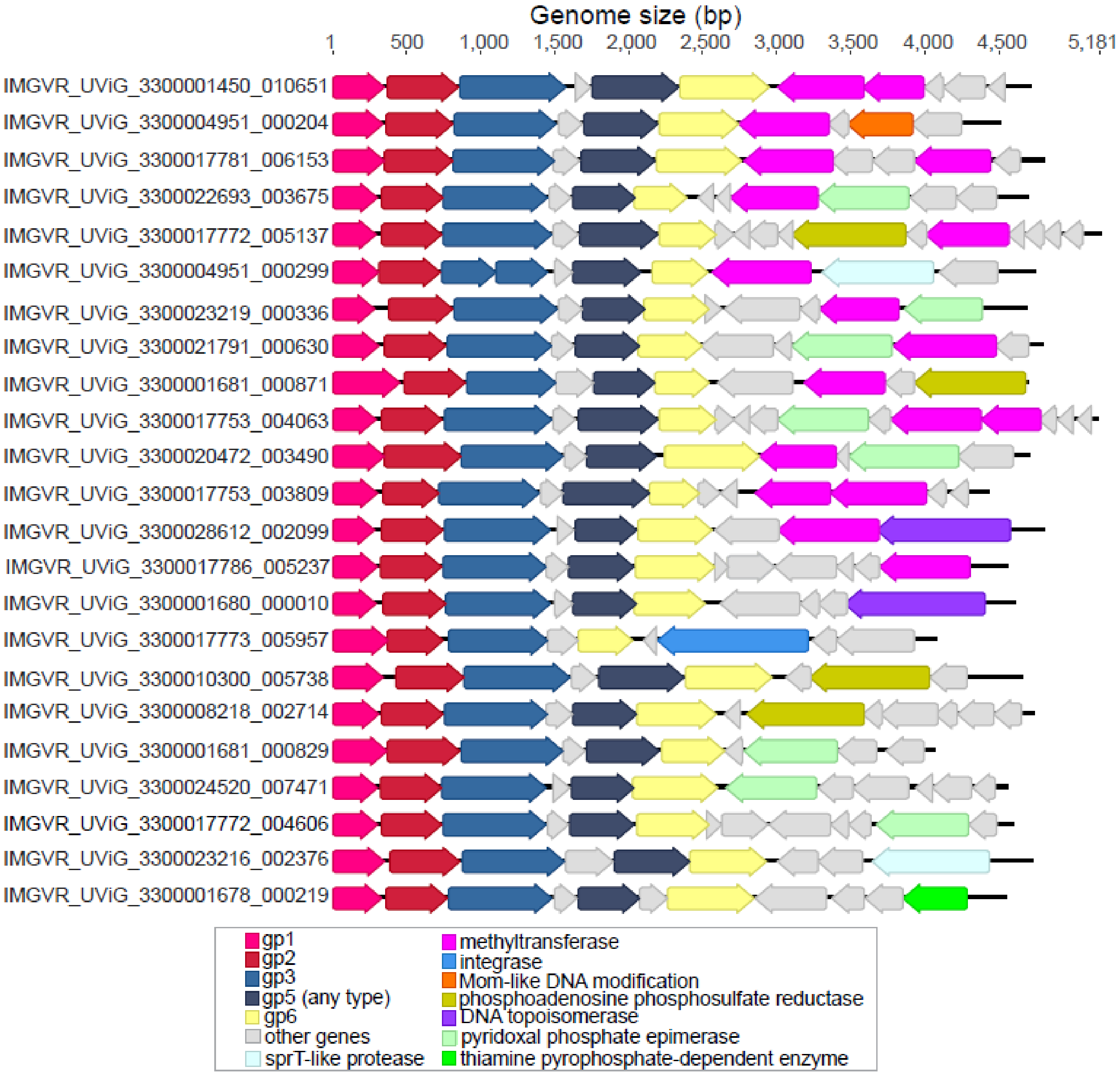

Figure S3: Gene content of select Charybdis elements, with focus on accessory genes. Arrows indicate genes. Circular genomes were arbitrarily linearized with gp1 as the 5’-end.

bioRxiv preprint doi: https://doi.org/10.1101/2025.08.03.668216; this version posted August 3, 2025. The copyright holder has placed this preprint (which was not certified by peer review) in the Public Domain. It is no longer restricted by copyright. Anyone can legally share, reuse, remix, or adapt this material for any purpose without crediting the original authors.

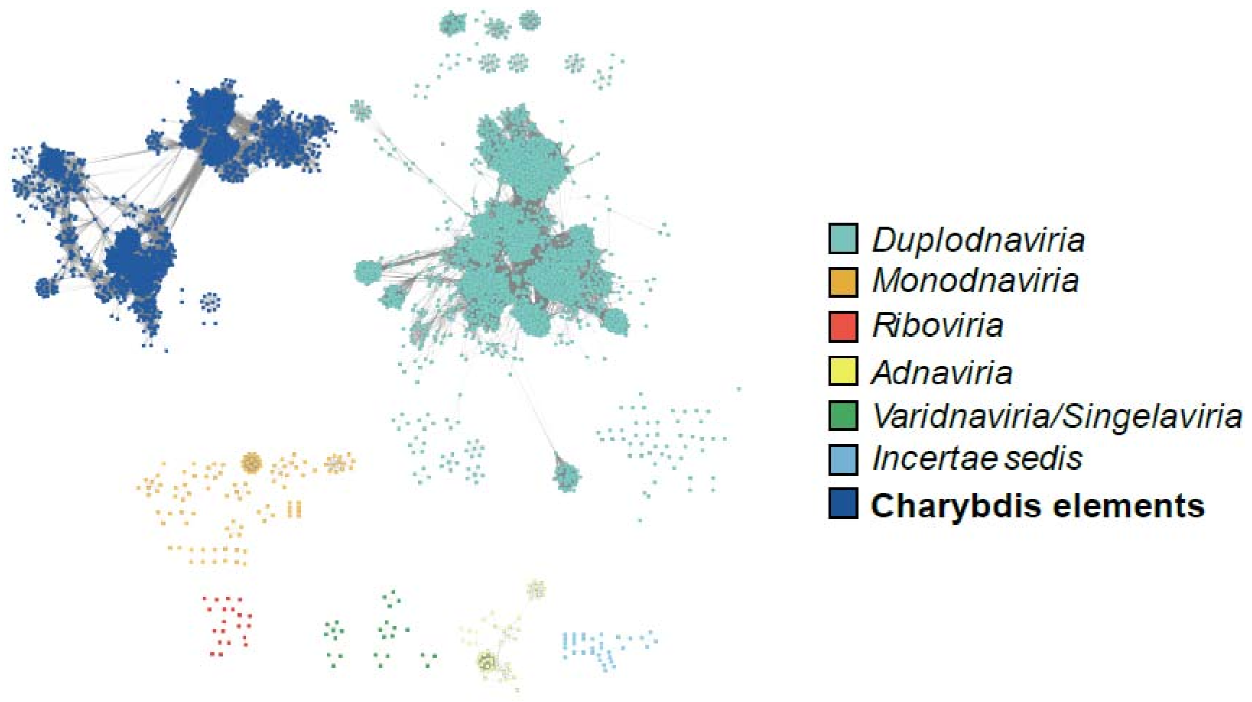

Figure S4: Protein sharing network of archaeal and bacterial viruses. Nodes represent individual genomes, connected by edges indicating shared proteins as calculated by vContact3 https://bitbucket.org/MAVERICLab/vcontact3/src/master/. Rectangles are coloured based on ICTV taxonomy.

bioRxiv preprint doi: https://doi.org/10.1101/2025.08.03.668216; this version posted August 3, 2025. The copyright holder has placed this preprint (which was not certified by peer review) in the Public Domain. It is no longer restricted by copyright. Anyone can legally share, reuse, remix, or adapt this material for any purpose without crediting the original authors.

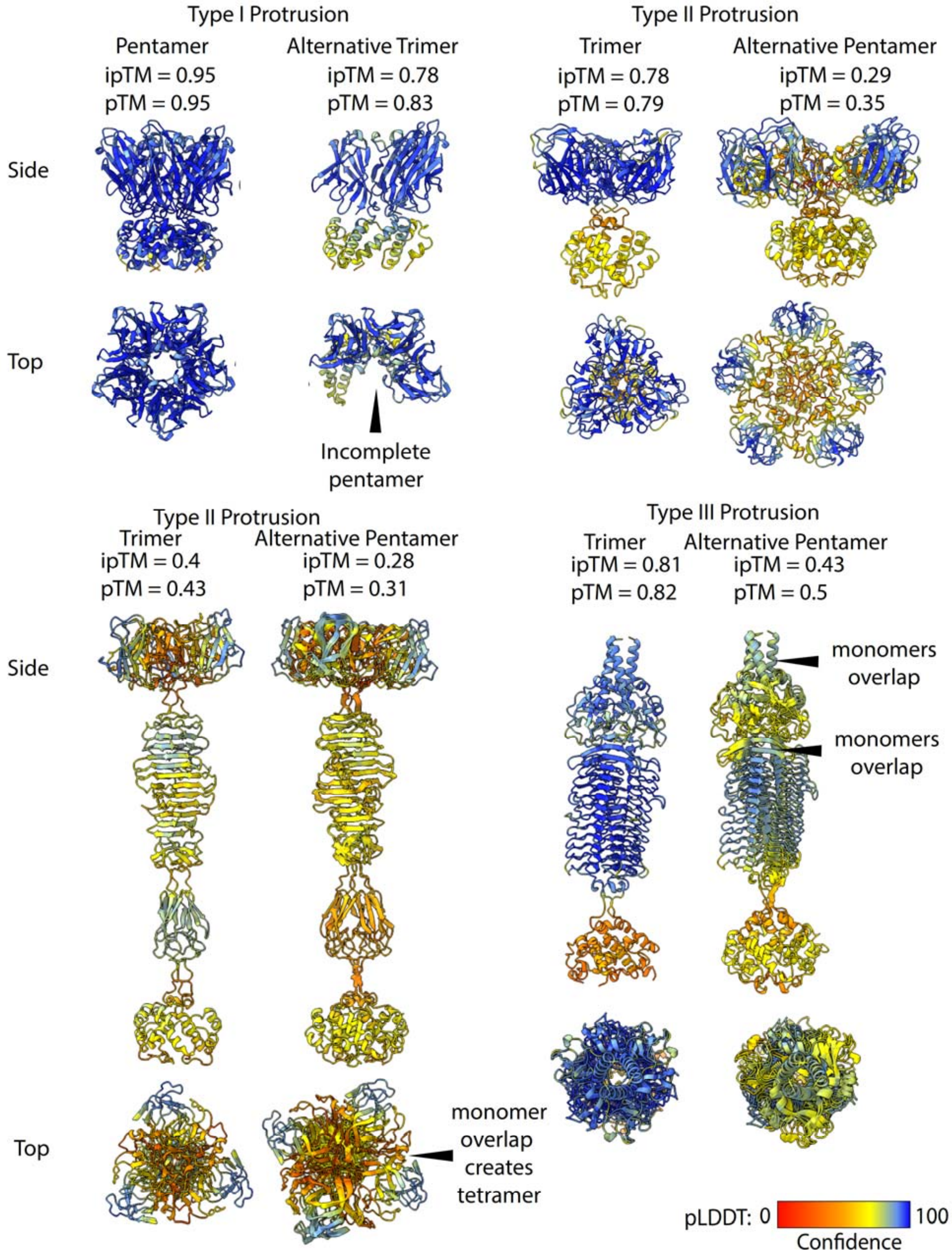

bioRxiv preprint doi: https://doi.org/10.1101/2025.08.03.668216; this version posted August 3, 2025. The copyright holder has placed this preprint (which was not certified by peer review) in the Public Domain. It is no longer restricted by copyright. Anyone can legally share, reuse, remix, or adapt this material for any purpose without crediting the original authors.

Figure S5: Alternative structural predictions and issues with Alphafold assemblies. Predictions are shown for IMGVR_UViG_3300004829_000422 (type I), IMGVR_UViG_3300024339_000993 and IMGVR_UViG_3300017782_005554 (type II) and (type III) IMGVR_UViG_3300025270_001813. pTM > 0.5 suggests that the predicted complex might be similar to the real structure. ipTM score 0.8 or above suggest high confidence predictions, below 0.6 suggest likely failed predictions. pLDDT (predicted local distance difference test represents a per-atom confidence score of predictions, with 100 (blue) representing the highest confidence and 0 (red) the lowest.

bioRxiv preprint doi: https://doi.org/10.1101/2025.08.03.668216; this version posted August 3, 2025. The copyright holder has placed this preprint (which was not certified by peer review) in the Public Domain. It is no longer restricted by copyright. Anyone can legally share, reuse, remix, or adapt this material for any purpose without crediting the original authors.

Supplementary Data

Supplementary datafile 1: Curated complete, circular Charybdis genomes analyzed in this study

Supplementary Table S1: Homology search results for all genes of genome depicted in Figures 1, S1

Supplementary Table S2: List of all Charybdis genomes in IMG/VR v4

Supplementary Table S3: Homology search results of exemplary gp5 protrusion types
